# A Novel Pool of Microparticle Cholesterol Is Elevated in Rheumatoid Arthritis but Not in Systemic Lupus Erythematosus Patients

**DOI:** 10.3390/ijms21239228

**Published:** 2020-12-03

**Authors:** Shuaishuai Hu, Brenton L. Cavanagh, Robert Harrington, Muddassar Ahmad, Grainne Kearns, Steve Meaney, Claire Wynne

**Affiliations:** 1 School of Biological and Health Sciences, College of Sciences and Health, Technological University Dublin—City Campus, D08 NF82 Dublin 8, Ireland; hushuaishuai87@gmail.com; 2 Environment, Sustainability and Health Institute, Technological University Dublin—Grangegorman Campus, D07 ADY7 Dublin 7, Ireland; 3 Cellular and Molecular Imaging Core, Royal College of Surgeons in Ireland, D02 YN77 Dublin 2, Ireland; brentoncavanagh@rcsi.ie; 4 Department of Rheumatology, Beaumont Hospital, D09 V2N0 Dublin 9, Ireland; robert.harrington86@gmail.com (R.H.); muddassara87@gmail.com (M.A.); grainnekearns3@gmail.com (G.K.)

**Keywords:** microparticles, cholesterol, systemic lupus erythematosus, rheumatoid arthritis, biomarker

## Abstract

Microparticles are sub-micron, membrane-bound particles released from virtually all cells and which are present in the circulation. In several autoimmune disorders their amount and composition in the circulation is altered. Microparticle surface protein expression has been explored as a differentiating tool in autoimmune disorders where the clinical pictures can overlap. Here, we examine the utility of a novel lipid-based marker—microparticle cholesterol, present in all microparticles regardless of cellular origin—to distinguish between rheumatoid arthritis (RA) and systemic lupus erythematosus (SLE). We first isolated a series of microparticle containing lipoprotein deficient fractions from patient and control plasma. There were no significant differences in the size, structure or protein content of microparticles isolated from each group. Compared to controls, both patient groups contained significantly greater amounts of platelet and endothelial cell-derived microparticles. The cholesterol content of microparticle fractions isolated from RA patients was significantly greater than those from either SLE patients or healthy controls. Our data indicate that circulating non-lipoprotein microparticle cholesterol, which may account for 1–2% of measured cholesterol in patient samples, may represent a novel differentiator of disease, which is independent of cellular origin.

## 1. Introduction

Microparticles are sub-micron, membrane-bound vesicles abundant in the circulation of many species [1] and form part of a spectrum of such particles from the exosomes to the apoptotic cellular remnants, collectively termed extracellular vesicles [2]. Although initially considered waste products, it is now evident that this family of circulating cellular fragments possess a range of different biological activities [3,4]. The specific activities are dictated by their internal cargo (e.g., nucleic acid, metabolites) and the protein and lipid components present in their membrane [5,6,7].

Release of microparticles is triggered by various physiological and pathological processes, which also influence their overall composition, and therefore function [2]. Pro-inflammatory cytokines, reactive oxygen species and coagulation factors drive the release of microparticles [8,9]. It is well-established that the number of microparticles present in the circulation of patients with autoimmune conditions such as rheumatoid arthritis (RA) and systemic lupus erythematosus (SLE) is significantly elevated [10]. These microparticles contain immunomodulatory molecules such as high-mobility group protein 1 (HMGB1) and can also form immune complexes, and therefore, influence inflammation, coagulation, antigen presentation and apoptosis [11]. Although the complete details of their involvement in disease is not yet understood, microparticles are considered to be important effectors of the overall pathology in autoimmune disorders [12,13].

Microparticle surface protein expression has been explored as a differentiating tool in autoimmune disorders where the clinical pictures can overlap. Anti-nuclear antibody positivity is identified in both SLE and RA, with both conditions being part of a spectrum of autoimmune connective tissue diseases. Joint involvement is a hallmark feature of RA; however, this is also commonly found in SLE. Rheumatoid Factors occur in up to 80% of people with RA [14] but are also found in up to 30% of people with SLE [15]. The erythrocyte sedimentation rate (ESR) also tends to be raised in both conditions. Indeed, it has been long debated whether these commonalities reflect the presence of a single disease with features of both [16,17], or the occurrence of two distinct diseases in an individual [18]. Extensive laboratory investigations are often required to support the clinical diagnosis and microparticles may present an accessible route to distinguish between such autoimmune conditions in a clinical context.

Cholesterol is a key component of vertebrate cellular membranes and is present in a number of functionally distinct pools within these membranes [19]. As they are membrane bound, all extracellular vesicles, including microparticles, contain phospholipids and cholesterol in their limiting membrane [20,21,22]. Such lipids are essentially insoluble in the plasma and must be carried by lipoproteins under physiological conditions. Microparticle-associated cholesterol thus represents a hitherto unexplored pool of cholesterol with unknown function and properties. Notably, as they are produced by different cellular processes, microparticles lack integral structural apolipoproteins (i.e., apolipoproteins A1 and B), and thus, will be unable to interact with lipoprotein receptors and integrate directly into normal lipoprotein homeostasis.

Studies of microparticle cholesterol have been hampered by difficulties in separating microparticles from cholesterol-rich lipoproteins which share similar biophysical properties [20]. However, using a combination of size-exclusion methods with ultracentrifugation has emerged in recent years which facilitate the isolation of lipoprotein-deficient microparticles [23]. In this paper we describe the application of these methods to characterise microparticle cholesterol in healthy controls, RA and SLE patients.

## 2. Results

### 2.1. Participant Details

Due to the nature of the disease, RA patients (median age of 60) were significantly older than SLE patients (median age of 43) (*p* < 0.05) or controls (median age of 45) (*p* < 0.01). Female/male ratios between healthy controls (10/3) and patients (RA:11/4, SLE:10/2) were equivalent. The mean disease duration between patients was comparable, with a median value of 10 and 11 years for SLE and RA, respectively (Table 1). There were no significant differences in total plasma triglycerides (Supplementary Figure S1) between each group, although there were slightly more RA patients with triglycerides outside of the reference interval. Despite the observed age difference in the participant cohorts, there is no difference in total cholesterol, an expected confounder in relation to any cholesterol-related measure. In the SLE group, three patients were treated with Mycophenolate, three with Prednisolone, six with Plaquenil, one with Azathioprine and one with Valacyclovir. In the RA group, nine patients were treated with Methothrexate, four with Hydroxychloroquine, two with Sulfasalazine and one with Etanercept.

### 2.2. Biophysical Characteristics of Microparticles

**Figure 1:**
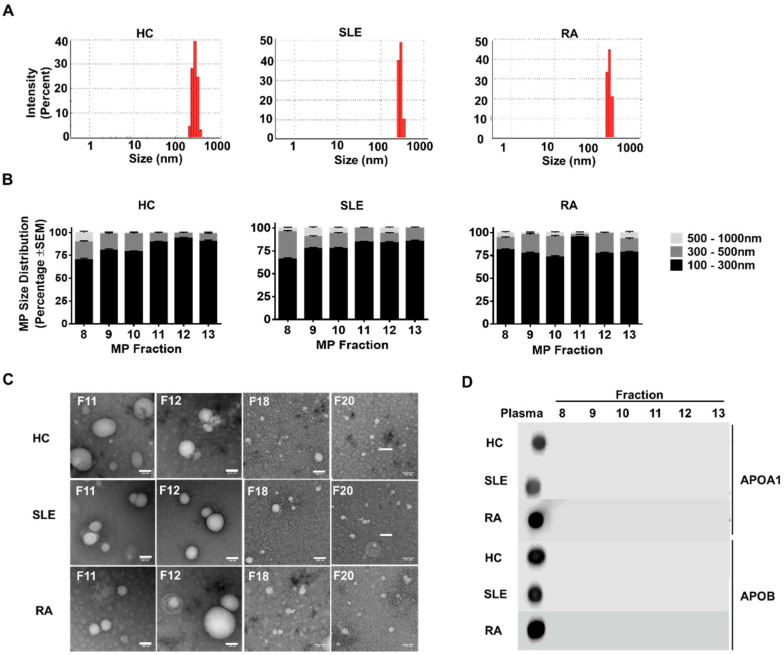
Microparticles from different groups share similar biophysical characteristics. (**A**) Representative sizing graphs from healthy control (HC), systemic lupus erythematosus (SLE) and rheumatoid arthritis (RA) microparticles (MP) using dynamic light scattering (DLS). The intensity (percentage) of particles present at a particular size was measured. In all cases, the size of particles in fractions 8–13 ranged from 100 to 1000 nm. (**B**) MP size distribution as determined by DLS. In all cases, the majority (>60%) of MP present in fractions 8–13 were between 100 and 300 nm (black bar), with approximately 25% of MP being between 300 and 500 nm in size (dark grey bar) and the remainder falling into the 500–1000 nm sizing bracket (light grey bar). Data are expressed as mean + SEM. (**C**) Representative TEM images from HC, SLE an RA fractions 11, 12, 18 and 20. In all cases, generally, particles ≥ 100 nm in size are evident in fractions F11 and 12 and not in later fractions 18 and 20. Scale bar = 100 nm. (**D**) Lack of APOA1 and APOB immunoreactivity in fractions 8–13. Plasma was used as a positive control.

Using the size-exclusion method noted above, 30 fractions were isolated from each sample. As noted above, pilot studies in our laboratory had identified fractions 8–13 as being microparticle-enriched, in accordance with literature data [24]. Zetasizer analysis revealed that in all cases, the size of particles in fractions 8–13 ranged from 100 to 1000 nm (Figure 1A). The average size of microparticles in fractions 8–13 from control or patient groups were comparable, with on average 60–70% of all isolated particles being between 100 and 300 nm, 20–30% being between 300 and 500 nm and less than 10% being greater than 500 nm in size (Figure 1B). No statistically significant differences were observed between the different participant groups. Although we did note an unusual prevalence of smaller particles in fraction 11 from RA patients (Figure 1B), there was no significant difference in microparticle size distribution between the participant groups. Transmission electron microscopy of fractions 8–13 revealed vesicles consistent with microparticle structure, while examination of fractions 18–20 (known to be lipoprotein enriched) revealed abundant smaller particles (less than 100 nm) (Figure 1C). Routine dot-blotting for apolipoproteins A1 and B_100_ did not reveal significant lipoprotein content in these fractions (Figure 1D). There was no significant difference in the protein content of the pooled fractions 8 to 13 between healthy controls, SLE and RA patients (Supplementary Figure S2).

### 2.3. Immunophenotyping of Microparticles

**Figure 2:**
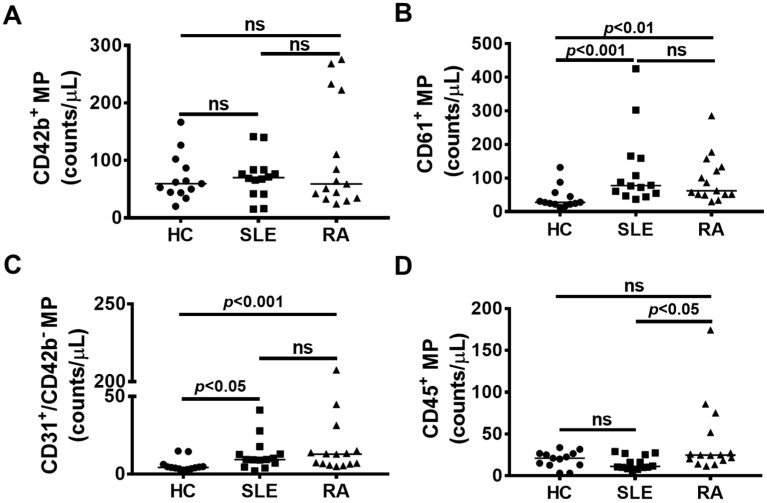
Autoimmune patients have increased platelet-, endothelial- and leucocyte-derived microparticles (MP). Fractions 8 to 13 were pooled and flow cytometry was used to determine the cell of origin of MP. (**A**) There was no significant difference in the quantity of CD42b (platelet GP1b)-derived MP between all groups. (**B**) Both patient groups had increased amounts of (**B**) CD61 (platelet GpIIIa)-derived and (**C**) CD31^+^CD42^−^ (endothelial)-derived MP compared to healthy controls (HC). (**D**) CD45 (leucocyte)-derived MP were increased in rheumatoid arthritis (RA) patients compared to both HC and systemic lupus erythematosus (SLE) patients. The horizontal bar indicates the median; a *p* value < 0.05 was considered significant. ns = not significant. While data are shown here as the pooled lipoprotein-deficient microparticles, profiling of individual fractions is show in Supplementary Figure S3.

Flow cytometry analysis on individual fractions 8–13 identified a greater amount of platelet (CD61^+^)-derived microparticles in the circulation of RA and SLE patients (*p* < 0.01) compared to healthy controls (Figure 2B). There was no difference in the content of CD42b^+^ platelet-derived microparticles (Figure 2A). Both RA and SLE patients contained more endothelial cell (CD31^+^, CD42b^-^)-derived microparticles than controls (*p* < 0.001 and *p* < 0.05, respectively) (Figure 2C). Neither SLE nor RA patients contained significantly more CD45^+^ (leucocyte)-derived microparticles compared to controls, although RA patients were shown to have significantly more compared to SLE patients (*p* < 0.05) (Figure 2D). We did not observe a difference in the number of CD42b platelet (glycoprotein Ib)-derived microparticles between groups. However, we did observe a difference in CD61 (glycoprotein IIIa)-derived microparticles, most likely due to the contribution of non-platelet microparticles (e.g., endothelial-, myeloid- and erythroid-derived particles) to the overall count.

### 2.4. Microparticle Cholesterol Is Elevated in Rheumatoid Arthritis but Not in Systemic Erythematosus Patients

**Figure 3:**
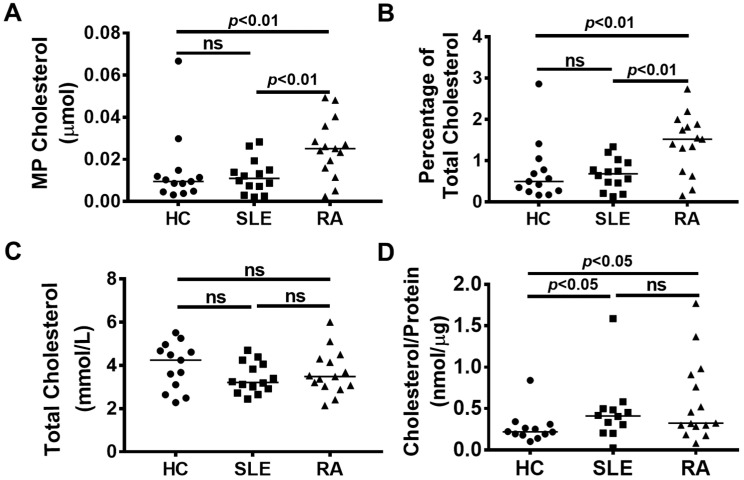
Rheumatoid Arthritis (RA) microparticles (MP) contain significantly more cholesterol than healthy control (HC) and systemic lupus erythematosus (SLE) MP. (**A**) Fractions 8 to 13 were pooled and the cholesterol content of MP was determined. RA MP contained significantly more cholesterol than both HC and SLE MP. (**B**) MP cholesterol was expressed as a percentage of the total plasma cholesterol in the HC and patient group. This proportion of cholesterol was significantly increased in RA patients compared to both HC and SLE patients. (**C**) There was no significant difference in the total cholesterol concentration between any group, with all participants having a total cholesterol measurement within the normal range. (**D**) The MP cholesterol to MP protein ratio was determined for all groups. The horizontal bar indicates the median; a *p* value <0.05 was considered significant. ns = not significant.

**Figure 4:**
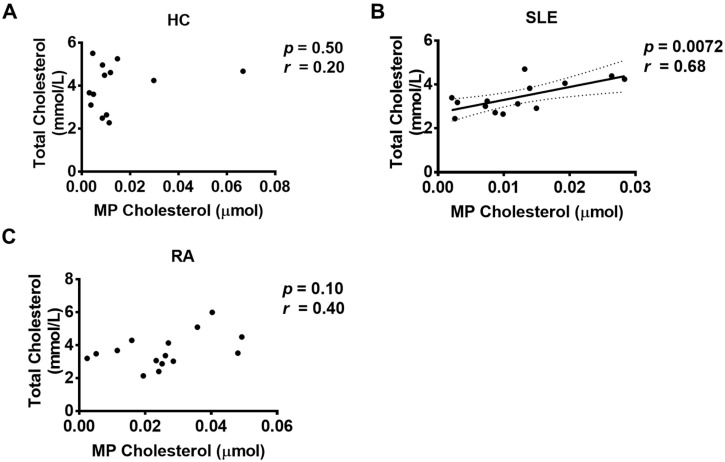
Correlations between total plasma cholesterol and microparticle (MP) cholesterol. There were no significant correlations between total plasma cholesterol and MP cholesterol from (**A**) healthy controls (HCs) or (**C**) Rheumatoid Arthritis (RA) patients, while there was a significant correlation in the case of (**B**) Systemic Lupus Erythematosus (SLE) (*p* < 0.01).

Total microparticle cholesterol (i.e., the sum of cholesterol present in fractions 8–13 for each participant) was significantly elevated in RA patients but not SLE patients compared to controls (Figure 3A). Microparticle cholesterol in samples from RA was thus significantly different from both controls and SLE patients (*p* < 0.01 for each comparison, see Figure 3A). To correct for variations in total plasma cholesterol between the patients, we expressed the microparticle cholesterol as a percentage of the total plasma cholesterol (i.e., moles of microparticle cholesterol per mole of plasma cholesterol) (Figure 3B). This correction would be expected to compensate for any undetected residual lipoproteins below our limit of detection. Differences remained significant after this transformation. There were no significant differences in total plasma cholesterol (Figure 3C) between the different groups and there was no significant correlation between microparticle cholesterol and participant age, although there was a tendency towards an age-related increase for SLE patients (Supplementary Figure S4). Normalisation of microparticle cholesterol to protein did not impact the difference between controls and RA patients, although the ability to distinguish between SLE and RA patients was lost (Figure 3D). There was no significant correlation between total plasma cholesterol and microparticle cholesterol from RA patients or controls, while there was a significant correlation in the case of SLE (*p* < 0.01) (Figure 4). These results indicate that the pool of microparticle cholesterol is not directly derived from that of plasma cholesterol in the cohorts studied here.

## 3. Discussion

Microparticles have been explored in relation to their potential as mediators, therapeutic tools and biomarkers in different rheumatic diseases [25,26]. Our study aimed to characterise microparticle cholesterol in patient groups where the absolute number of microparticles is known to be increased, with a view towards understanding the potential contribution of microparticle cholesterol to cardiovascular risk in RA patients. We included SLE patients as a related inflammatory disease control group and also to provide the opportunity if microparticle cholesterol could distinguish between two diseases which share many clinical signs [18].

Our immunophenotyping findings are consistent with other investigators who have reported elevated numbers of platelet (CD61^+^)- and leucocyte-derived (CD45^+^) microparticles in the circulation of RA and SLE patients compared to healthy controls [10,27,28]. While CD45 positivity was not sufficient to separate healthy controls from patients in our study, it was able to distinguish between RA and SLE patients. As previously reported for SLE [29], the numbers of endothelial cell-derived microparticles (CD31^+^CD42b^−^) were also increased in our SLE cohort. Surprisingly, we also identified a similar increase in CD31^+^CD42b^−^ microparticles in our RA patients. While this result highlights their potential use in the identification of RA, it requires confirmation in other patient cohorts. In addition, it highlights the challenges of identifying suitable biomarkers for these conditions.

Although they are well-recognised as being membrane-bound, and thereby lipid vesicles, the cholesterol content of circulating microparticles is poorly studied, which limits its potential use as a biomarker. The potential impact of microparticle preparations with cholesterol-rich lipoproteins must also be considered, and steps must be taken to ensure that microparticle preparations are essentially lipoprotein free. As the Svedberg coefficients of microparticles and lipoproteins overlap, it is extremely difficult to ensure that there is separation when using ultracentrifugation-based methods to isolate microparticles [23]. It is also difficult to interpret the microparticle lipid content in such studies where there may be contamination with plasma lipoproteins and their constituent proteins [5]. Other separation methods, such as size exclusion chromatography, have proved more effective in producing microparticle preparations with minimal lipoprotein content, which are thus suitable for studies of microparticle cholesterol content [24].

As part of quality control processes, we routinely tested our microparticle-enriched fractions with dot-blots against apolipoprotein A1 and B, the major apolipoproteins of the human circulation and the core structural proteins of HDL and LDL, respectively. As highlighted in Figure 1, we did not detect any immunoreactivity against either of these proteins in fractions 8–13. Detailed mapping across all fractions of a control donor using antibodies to apolipoproteins A1, B, E, D, H, M and J revealed only trace amounts of reactivity in fractions 12 and 13, amounting to less than 0.1% of the total plasma apolipoprotein B content. Given that each LDL particle contains on average of 2100 ± 400 molecules of cholesterol, and assuming that each LDL particle contains one apolipoprotein B molecule, it may be calculated that for a concentration of 1000 ng/mL of apolipoprotein B in a solution (i.e., 181 pM), a corresponding concentration of approximately 360 nM of cholesterol would be expected, i.e., in approximately 300 pmoles/microparticle fraction. This is significantly lower than the amount of cholesterol detected in the patient and control samples, which are typically in the nanomolar range, indicating that the cholesterol detected here is not significantly influenced by lipoprotein contamination in the preparations used. It should be noted that the estimated cholesterol/LDL particle is averaged from some 3066 patients [30], taken from the Framingham heart study with varying levels of hypertriglyceridemia and so likely represents an overestimation compared to the current cohort. In addition, the values of 1000 ng/mL used for calculations is an overestimate and well within the sensitivity of the dot blot method used here.

In further support of this view, in both healthy controls and RA patients, there is a lack of correlation between total plasma cholesterol and microparticle cholesterol, indicating that the pool of cholesterol associated with microparticles is distinct from that of lipoproteins. Thus, the observed elevation in microparticle cholesterol in RA samples is not significantly influenced by circulating lipoproteins. This was not the case in SLE patients, which is consistent with the observed data that microparticle cholesterol can distinguish SLE and RA patients. At the current stage of knowledge, and acknowledging the small size of this study, we cannot exclude that there is some contribution of lipoprotein cholesterol to the total microparticle cholesterol in various patient cohorts. In addition, the independence of the microparticle cholesterol pool has not been empirically assessed—this will be an essential piece of knowledge to understand the utility of this measure in clinical practice and for the understanding of cholesterol physiology.

It is also important to account for the potential contribution of clinical therapeutics which may influence cholesterol homeostasis, for example methotrexate. It has been described that methotrexate treatment may lead to an acute increase of total cholesterol by approximately 1 mM [31]. Although the detailed kinetics of this increase are incompletely described, the median treatment duration in our cohort is many times the previously described acute induction phase of 24 weeks. We, thus, anticipate that methotrexate-induced changes will have stabilised in these patients. Given the poor correlation between total (lipoprotein) and microparticle cholesterol, such changes would not in any case be expected to significantly influence the current results. Expression of microparticle cholesterol as a percent of total cholesterol should compensate for any changes in serum cholesterol—as Figure 3 highlights, the observed differences remain after this normalization.

As noted in Figure 3, normalization to microparticle protein content influenced the statistical significance of the cohort comparisons. While some RA patients have a higher MP cholesterol to MP protein ratio than others, there was no obvious difference between the RA patients as a result of methotrexate treatment, with a significant overlap between the two populations. However, the small size of the treated and untreated populations cannot exclude a potential effect of drug treatment. These data also indicate that the protein composition, and by extension the functional proteome of the microparticles, may influence their cholesterol content. This may be related to the route of formation of the microparticles—previous studies on microparticle cholesterol [32,33] have indicated that microparticle membranes may be significantly enriched in cholesterol, presumably related to the way in which these particles are formed and released. It can be speculated that microparticles from RA patients originate from cells which have an elevated membrane cholesterol content or from regions of the cell with greater cholesterol content. However, at the current state of knowledge it is difficult to explain the physiological reason for the changes in microparticle cholesterol between the different cohorts.

Erum et al. have reported that dyslipidaemia is observed in active RA disease, with patients often having low cholesterol levels yet high cardiovascular disease risk [34]. The paradoxical lipid changes observed in RA is not fully understood. This study provides new insights which may contribute to explaining this finding. We estimate that the cholesterol contained in microparticles may represent a minor but significant pool of cholesterol (1–2% of total cholesterol) in the circulation. Importantly, the metabolic fate of microparticle cholesterol has not, to the authors’ knowledge, been elucidated. It is reasonable to assume that, given the lack of key apolipoproteins, microparticle cholesterol does not integrate into normal lipoprotein homeostasis, and thus, may be taken up aberrantly in cells with unknown consequences on cellular cholesterol homeostasis. The combination of the bioactive nature of microparticles and their role as a cholesterol delivery system may be a potent combination, which contributes to the enhanced cardiovascular disease risk in RA patients.

In addition to these new insights into microparticle cholesterol, our studies of SLE and RA also demonstrates for the first time that microparticle cholesterol in RA patients is significantly increased compared to healthy controls and SLE patients, suggesting its potential use for the diagnosis of RA and to distinguish RA from SLE. We would note that this is an initial exploration of a previously unknown pool of cholesterol, and thus, represents a pilot study. We encourage other researchers to carry out similar studies in other cohorts and patient types to validate and extend our data. Such studies are ongoing in our laboratory.

## 4. Materials and Methods

### 4.1. Ethical Approval

All subjects gave their written informed consent for inclusion before they participated in the study. The study was conducted in accordance with the Declaration of Helsinki, and the protocol was approved by the Ethics Committees of both Beaumont Hospital (reference 16-65) and Technological University Dublin (reference REC-16-49).

### 4.2. Study Population

RA and SLE patients were recruited from routine outpatient clinics in Beaumont Hospital. Clinical information was collected via chart review. All patients are Irish and the diagnosis of RA or SLE was based on the European League against Rheumatism/American College of Rheumatology and the Systemic Lupus International Collaborating Clinics (SLICC) criteria for RA and SLE, respectively [35,36]. Controls were recruited from the staff and student body of Technological University Dublin.

### 4.3. Collection and Pre-Processing of Participant Samples

Blood samples were collected into two 3.5 mL sodium citrate vacutainer tubes, using a 21-gauge needle by trained medical staff or phlebotomists. All patient and control samples were processed by the same operator between 4 and 6 hr after sample collection. Platelet-poor plasma (PPP) was obtained by centrifugation at 1500× *g* for 10 min at room temperature, followed by a second centrifugation at 13,000× *g* for 30 min at 4 °C to obtain platelet-free plasma (PFP). The PFP from each participant was aliquoted into 1.5 mL microcentrifuge tubes and stored at −80 °C until required.

### 4.4. Isolation of Lipoprotein-Deficient Microparticles

Microparticles were isolated as described by Boing et al. [24]. Briefly, an in-house column of Sepharose CL-2B (10 mL column volume) was poured and conditioned with three column-volumes of running buffer (phosphate-buffered saline, 137 mM NaCL, 2.7 mM KCl, 10 mM NA_2_HPO_4_, 1.8 mM KH_2_PO_4_, pH 7.4 containing 0.32% (*w/v*) trisodium citrate). PFP (0.5 mL) was applied to the column and 30 microparticle fractions (~450 μL) were collected via elution using running buffer. Fractions were stored at –80 °C until required. Each column was used only once.

Based on previous data and previous experience in our laboratory, fractions 8–13 were known to represent the microparticle containing fractions, with apolipoprotein-B and apolipoptrotein-A containing lipoproteins peaking at fraction 23 and 30, respectively. Dot blots for apolipoproteins were routinely used to quality control the fractions for lipoprotein contamination in fractions 8–13, as described below.

Briefly, 2 µL of each fraction sample from platelet concentrates or donor plasma was spotted onto a nitrocellulose membrane. The membrane was blocked with 5% bovine serum albumin (BSA) for 1 h at room temperature, followed by incubation with a primary antibody for 1 h at room temperature. All primary antibodies used were from Mabtech AB (Nacka Strand, Sweden) and diluted as follows in 5% BSA: APOA1 (HDL-44-biotin, 3710-6) (1:1000), APOB (LDL-11-biotin, 3715-6) (1:5000), APOH (H464-biotin, 3711-6) (1:1000), APOJ (J100-biotin) (1:1000), APOM (M5-biotin) (1:1000), APOD (D544-biotin) (1:1000) and APOE (mAbE887, 3712-6) (1:1000). After washing three times with Tris-buffered saline with 0.1% tween-20 (TBS-T), the membrane was incubated with an avidin-biotinylated secondary antibody (Vectastain AB Kit, Catalogue Number PK-6100 Series, Burlingame, CA, USA) following the manufacturer’s instructions for 30 min at room temperature. The membrane was washed three times in TBS-T and incubated with ECL reagent for 1 min, followed by analysis of chemiluminescent signal using the LI-COR Digital imaging system (LI-COR Biotechnology, Cambridge, UK). Any sample which displayed a signal in any of the tested fractions was considered to have failed in the separation process, and a fresh sample was separated.

### 4.5. Characterisation of Microparticle Size

MP size was determined using the Zetasizer ZS (Malvern Panalytical, Malvern, UK), which estimates the size of particles by measuring their speed as they undergo Brownian motion using dynamic light scattering, and which provides the relative distribution from the different size in terms of how many particles are present. Briefly, 50 µL samples from fractions 8–13 were diluted in 1 mL of phosphate-buffered saline (pH 7.4) containing 0.32% (*w/v*) trisodium citrate and were measured at 10 °C.

### 4.6. Estimation of Cholesterol Content in Lipoprotein-Deficient Microparticles

Cholesterol content was defined using the Amplex Red kit (A12216, Invitrogen, Gloucester, UK) according to the manufacturer’s instructions. This assay reacts with all 3b-hydroxy steroids. However, given that non-cholesterol sterols with this configuration are present at less than 0.01% of cholesterol in lipoprotein cores, we consider the assay specific for microparticle cholesterol. All assays were carried out in batch mode using an additional clinical-grade control.

### 4.7. Immunophenotyping of Isolated Microparticles

A 20 μL aliquot of each fraction 8–13 was stained with 5 μL of one of the following fluorescent monoclonal antibodies; PE-labelled anti-CD61 (555754, BD Biosciences, Wokingham, UK), PE-labelled anti-CD45 (555483, BD Biosciences), PE-labelled anti-31 (555446, BD Bioscience) and APC-labelled anti-CD42b (551061, BD Bioscience) for 45 min on ice. Thereafter, all samples were incubated at room temperature for 20 min, diluted with phosphate-buffered saline (pH 7.4) containing 0.32% (*w/v*) trisodium citrate and analysed by flow cytometry on the Accuri C6 (Accuri Cytometers, Wokingham, UK). For flow cytometry analysis of MP, events calibrated by internal standard beads (0.8 μm; Sigma-Aldrich, St. Louis, MO, USA) were identified in forward-scatter and side-scatter intensity dot representation, gated as the MP, and plotted on one-colour fluorescence histograms. For MP enumeration, Accuri C6 automatically calculated counts per µL for gated populations. The direct counts correlate highly (*r*^2^ = 0.999) with, and are as precise as, counts performed with counting beads. Unstained samples and isotype control antibodies were used as negative controls in all measurements. Additionally, fluorescence minus one (FMO) controls were used to identify CD31^+^ or CD42b^+^ MP in CD31/CD42b double staining analysis. Representative examples of flow cytometry results are shown in Supplementary Figure S5.

### 4.8. Electron Microscopy of Microparticle Samples

To visualise microparticles using transmission electron microscopy (TEM), 5 µL from each sample fraction was placed on a 200-mesh formvar and carbon coated copper grid (Ted Pella, Redding, CA, USA) for 10 min. Excess samples were wicked away before the grid was floated on a 20 μL drop of 25% Uranyl Acetate Alternative (Ted Pella) diluted in distilled water for 1 min. Excess stain was wicked away to yield a dry grid prior to image acquisition using a Hitachi H7650 transmission electron microscope (Maidenhead, UK) operating at 100 kV and side mounted camera.

### 4.9. Statistical Methods

One-way ANOVA testing followed by Tukey test was applied for normal distributed data, while one-way Kruskal-Wallis analysis followed by Mann-Whitney U test was applied to data which were not normally distributed. Univariate correlation analysis was performed with Spearman’s rank correlation. GraphPad Prism 7.0 (San Diego, CA, USA) was used for these analyses.

## Figures and Tables

**Table 1:** Demographic and clinical characteristics of study participants.

	HC	SLE	RA
***n***	13	14	15
Age (years) *	45 (26–58)	43 (31–75)	60 (33–75)
**Gender (F/M)**	10/3	10/2	11/4
Disease duration (years) *	N/A	10 (2–25)	11 (1–36)

HC; healthy controls, SLE; systemic lupus erythematosus, RA; rheumatoid arthritis, N/A; not applicable, * Median (minimum–maximum range).

## Supplementary Materials

The following are available online at https://www.mdpi.com/1422-0067/21/23/9228/s1.
