# Supplementary material for: A Novel Pool of Microparticle Cholesterol Is Elevated in Rheumatoid Arthritis but Not in Systemic Lupus Erythematosus Patients
Source: Int J Mol Sci. 2020 Dec 3;21(23):9228. doi: 10.3390/ijms21239228 (PMC7730612; doi:10.3390/ijms21239228)
Supplement: Supplementary file 1 [file ijms-21-09228-s001.pdf]

Supplementary Materials

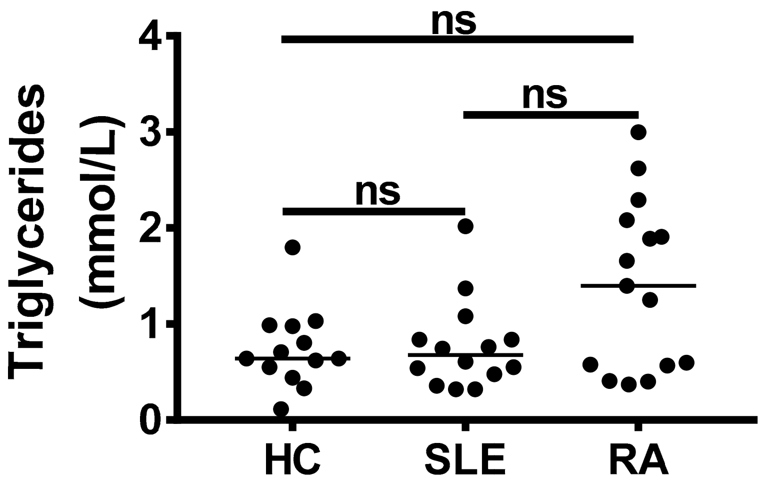

Figure S1. Triglyceride concentration. There were no significant differences in total plasma triglycerides between healthy controls (HC), systemic lupus erythematosus (SLE) and rheumatoid arthritis (RA) patients. Horizontal bar indicates median, ns = not significant.

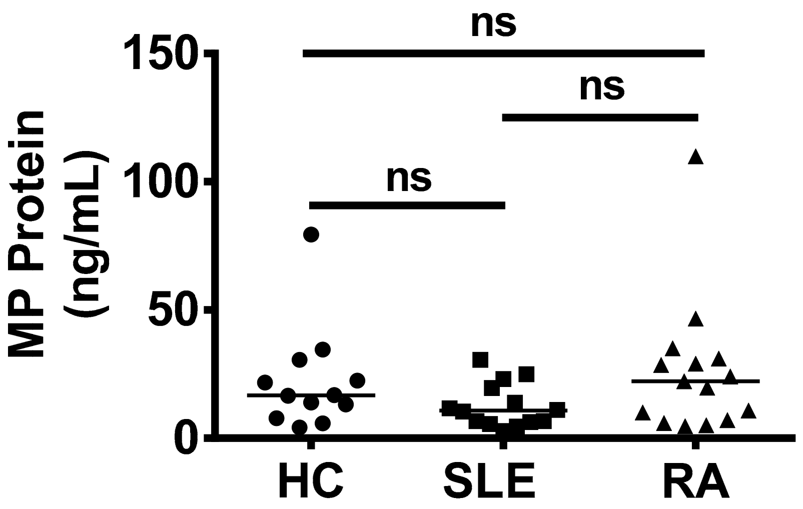

Figure S2. Microparticle protein concentration. There was no significant difference in protein concentration in pooled fractions 8 to 13 between healthy controls (HC), systemic lupus erythematosus (SLE) and rheumatoid arthritis (RA) patients. Horizontal bar indicates median, ns = not significant.

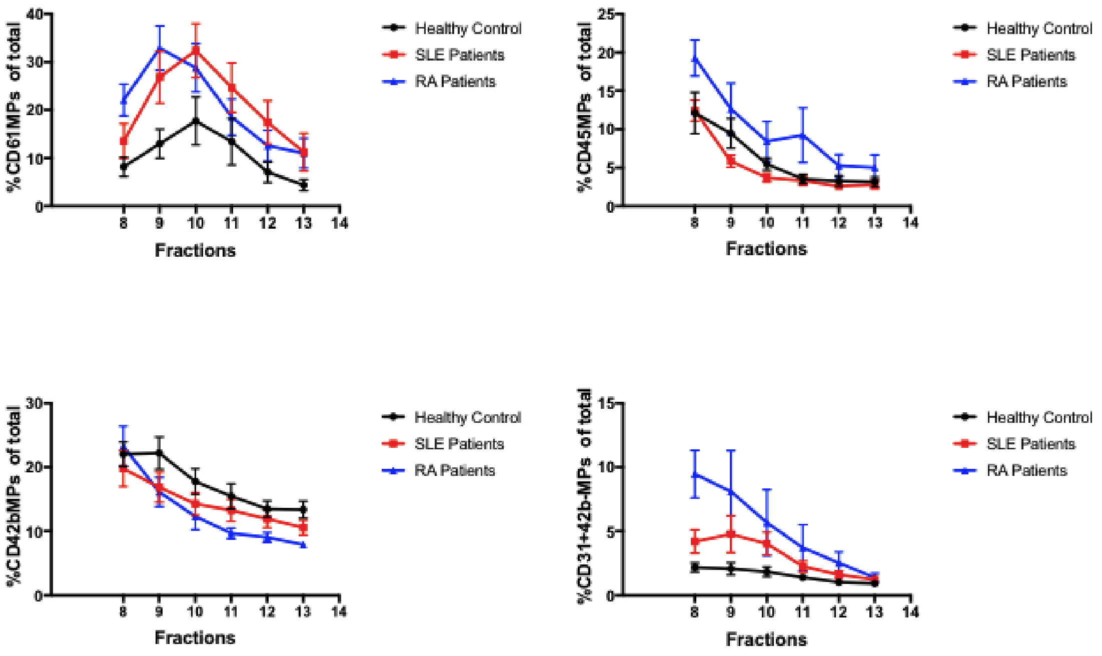

Figure S3. Immunophenotyping of individual microparticles (MP) from individual participant fractions. The Y-axis indicates the percent of all MP in that fraction which are positive for the indicated marker.

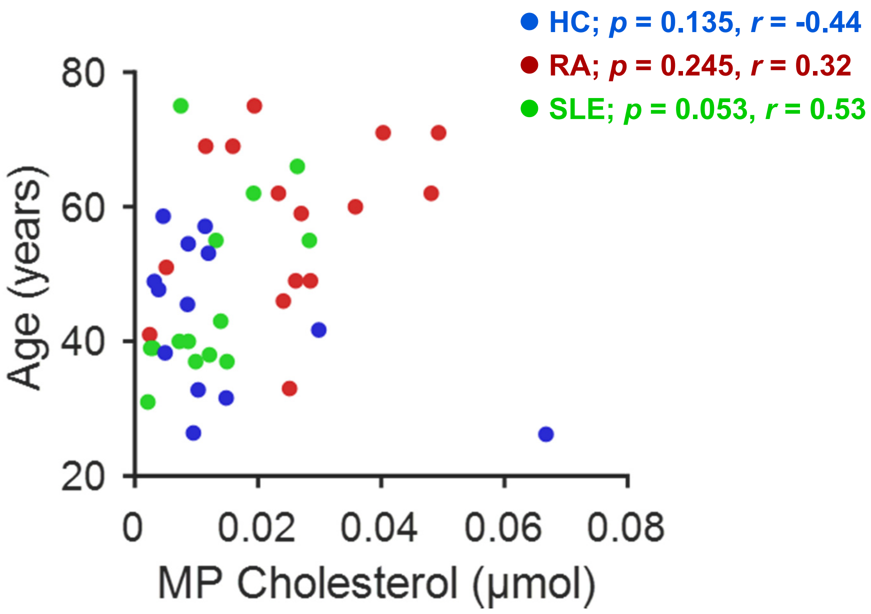

Figure S4. Correlation between age and microparticle (MP) cholesterol from different cohorts. No significant correlation is seen in any cohort, although there is a tendency to an age dependent increase in the systemic lupus erythematosus (SLE) group, HC; healthy controls, RA; rheumatoid arthritis

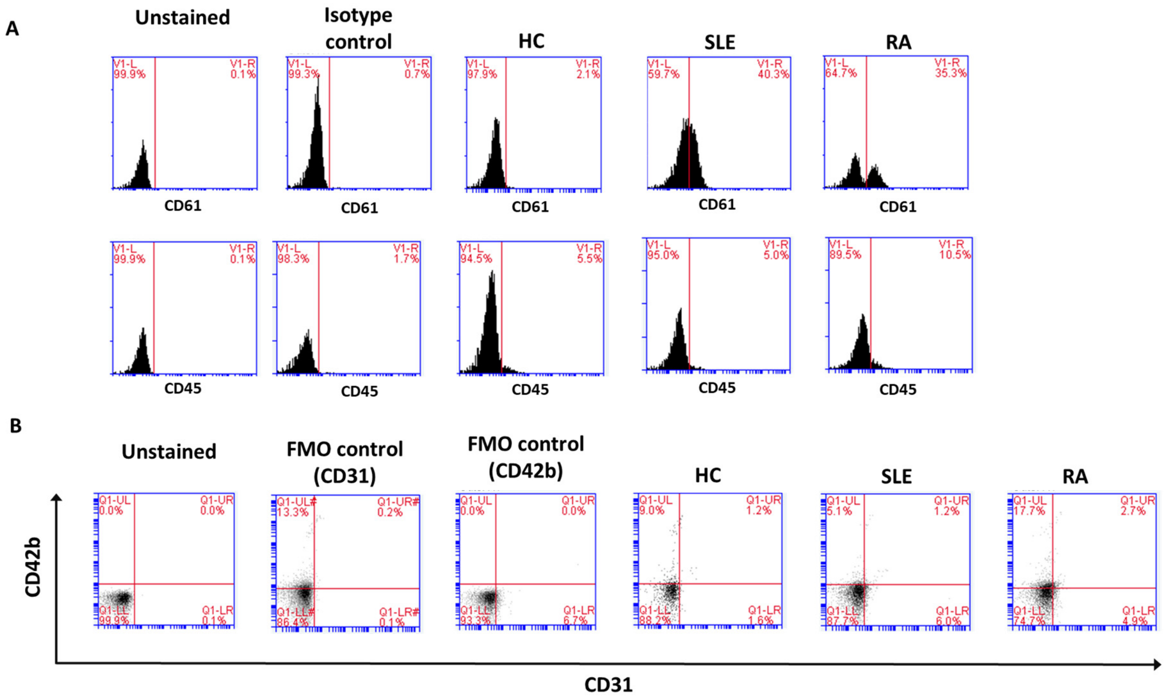

Figure S5. Representative example of flow cytometry analysis, including relevant controls, of microparticle fractions from different cohorts. (A) Staining for either CD61 or CD45 individually; (B) Dual staining for CD31/CD42b. HC; healthy controls, SLE; systemic lupus erythematosus, RA; rheumatoid arthritis, FMO; fluorescence minus one
